# Unveiling the Unexplored Multifactorial Potential of 5-Aminosalicylic Acid in Diabetic Wound Therapy

**DOI:** 10.3390/diseases12080172

**Published:** 2024-08-01

**Authors:** Bharat Kumar Reddy Sanapalli, Ashwini Deshpande, Vidyasrilekha Sanapalli, Dilep Kumar Sigalapalli

**Affiliations:** 1 Department of Pharmacology, School of Pharmacy and Technology Management, SVKM’s Narsee Monjee Institute of Management Studies (NMIMS) Deemed-to-be-University, Jadcherla 509301, Hyderabad, India; bharathsanapalli@yahoo.in; 2 Department of Pharmaceutics, School of Pharmacy and Technology Management, SVKM’s Narsee Monjee Institute of Management Studies (NMIMS) Deemed-to-be-University, Jadcherla 509301, Hyderabad, India; ashwinideshpande4@gmail.com; 3 Department of Pharmaceutical Chemistry, School of Pharmacy and Technology Management, SVKM’s Narsee Monjee Institute of Management Studies (NMIMS) Deemed-to-be-University, Jadcherla 509301, Hyderabad, India; 4 Department of Pharmaceutical Chemistry, Vignan Pharmacy College, Jawaharlal Nehru Technological University, Guntur 522213, Andhra Pradesh, India

**Keywords:** diabetic wounds, 5-aminosalicylic acid, inflammation, TGF-β activation, PPAR-γ activation, oxidation, re-epithelialization

## Abstract

Diabetic wounds (DWs) are considered chronic complications observed in patients suffering from type 2 diabetes mellitus (DM). Usually, DWs originate from the interplay of inflammation, oxidation, impaired tissue re-epithelialization, vasculopathy, nephropathy, and neuropathy, all of which are related to insulin resistance and sensitivity. The conventional approaches available for the treatment of DWs are mainly confined to the relief of wound pressure, debridement of the wound, and management of infection. In this paper, we speculate that treatment of DWs with 5-aminosalicylic acid (5-ASA) and subsequent activation of peroxisome proliferator-activated receptor gamma (PPAR-γ) and transforming growth factor beta (TGF-β) via the AhR pathway might be highly beneficial for DW patients. This estimation is based on several lines of evidence showing that 5-ASA and PPAR-γ activation are involved in the restoration of insulin sensitivity, re-epithelialization, and microcirculation. Additionally, 5-ASA and TGF-β activate inflammation and the production of pro-inflammatory mediators. Suitable stabilized formulations of 5-ASA with high absorption rates are indispensable for scrutinizing its probable pharmacological benefits since 5-ASA is known to possess lower solubility profiles because of its reduced permeability through skin tissue. In vitro and in vivo studies with stabilized formulations and a control (placebo) are mandatory to determine whether 5-ASA indeed holds promise for the curative treatment of DWs.

## 1. Introduction

Wound healing is a multistage process that requires cooperation between different cell types, cytokines, and growth hormones [1,2]. A thorough understanding of the pathophysiology of wound healing should aid in the development of effective therapeutic techniques. Hemostasis is the first step of wound healing; the primary response to damage includes vasoconstriction and platelet aggregation, which results in the development of a fibrin clot that inhibits additional blood loss while simultaneously serving as a temporary matrix for cell migration [1,2]. The next stage is inflammation, which occurs when neutrophils, macrophages, and lymphocytes penetrate the area and remove debris and microorganisms. TNF-α, IL-1, and TGF-β are inflammatory cytokines and growth factors that promote healing in the future [1,2]. During the proliferation stage, fibroblasts multiply and produce extracellular matrix (ECM) components such as collagen, which provide structural support. Angiogenesis is the process of producing new blood vessels that provide nutrients and oxygen to the repaired tissue. Keratinocytes migrate toward the wound surface, facilitating re-epithelialization. The final stage of remodeling comprises collagen fiber maturation and reorganization, which increases wound tensile strength. Myofibroblast wound contraction significantly reduces wound size [1,2]. Several therapeutic methods are available for wound management, including wound dressings [3], growth factors [4], topical antibiotics [5], antiseptics [5,6], negative pressure wound therapy, skin replacements [7], and skin grafts [7]. Despite recent advances, the treatments indicated above have drawbacks, such as limited availability, high cost, infection risk, and poor efficacy in chronic wounds [5,6,7,8]. These limitations underscore the need for more effective therapeutic strategies.

Diabetic wounds (DWs) are chronic or nonhealing wounds, particularly diabetic foot ulcers (DFUs), which present unique challenges due to the underlying pathophysiology of diabetes [9]. According to statistics from the International Diabetes Federation, 25% of patients with diabetes tend to develop DWs in their lifetime [10]. The major problem associated with DWs is impaired inflammation [11]. Patients often exhibit prolonged and exaggerated inflammatory responses, leading to a delayed transition to the proliferative phase. These include reduced angiogenesis, in which hyperglycemia impairs the formation of new blood vessels, limiting oxygen and nutrient supplies to the wound site. Additionally, there is ECM dysregulation, where altered collagen deposition and increased matrix metalloproteinase (MMP) activity contribute to ECM degradation and poor wound strength [11]. Furthermore, neuropathy and ischemia in diabetic neuropathy and peripheral arterial disease result in decreased sensation and blood flow, increasing the risk of wound formation and delayed healing. Exudate control, wound offloading, surgical debridement, dressing to maintain a moist wound environment, vascular assessment, glucose monitoring, and infection monitoring are some of the standard treatment procedures for managing DWs [12,13,14]. However, the aforementioned treatments, while focusing on wound debridement, infection prevention, and control, were unable to address all of these prerequisites due to the multifactorial nature of the disease [15]. Given the high prevalence and morbidity associated with diabetic wounds, there is an urgent need for novel therapies that can address specific pathophysiological challenges. There are currently few drugs that can be used to treat DWs. Some ideas for future treatments are to target multipronged molecular pathways that play a part in inflammation, angiogenesis, and ECM remodeling, as well as to use new technologies such as gene therapy, stem cell therapy, and biomaterials. We believe that 5-aminosalicylic acid (5-ASA), also known as mesalazine or mesalamine, an aminosalicylate anti-inflammatory medication, could benefit individuals with DWs.

## 2. Hypothesis

**Figure 1:**
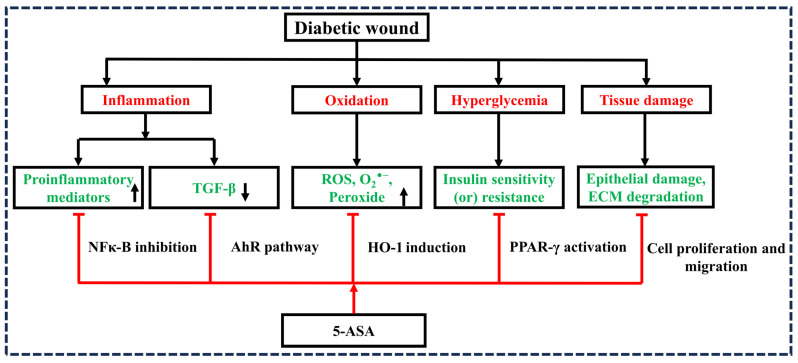
Schematic representation of diabetic wound inhibition by 5–aminosalicylic acid. Note: transforming growth factor beta (TGF–β), reactive oxygen species (ROS), oxygen free radical (O_2_^−^), extracellular matrix (ECM), nuclear factor-kappa B (NF–κB), heme oxygenase–1 (HO–1), peroxisome proliferator–activated receptor–γ (PPAR–γ), 5–aminosalicylic acid (5–ASA), inhibition (⊤).

Our hypothesis incorporates significant implications for treatment modalities based on the following factors. Importantly, 5-ASA has substantial effects on the regulation of inflammation [14,16], antioxidation [17], re-epithelialization [18,19], the activation of peroxisome proliferator-activated receptor gamma (PPAR-γ) [20,21], and transforming growth factor beta (TGF-β) [22], which play vital roles in wound healing progression (Figure 1). However, the exact mechanism of action of 5-ASA is not entirely known. Several in vitro and in vivo screening studies need to be performed to understand the precise role of 5-ASA in the treatment of DWs.

## 3. Evaluation of the Hypothesis

### 3.1. 5-ASA and Inflammation

**Figure 2:**
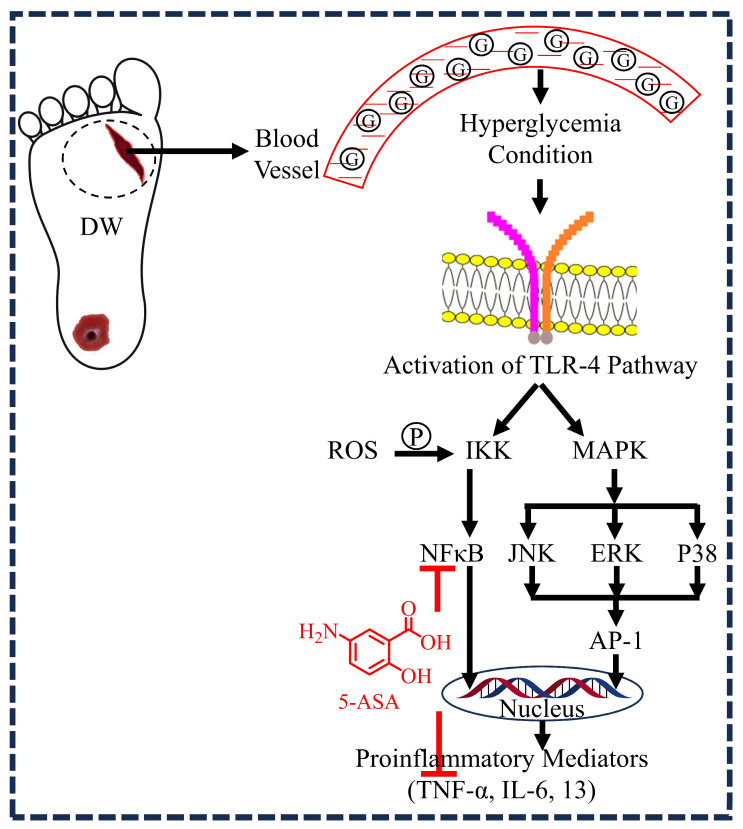
5-aminosalicylic acid (5-ASA) primarily inhibits nuclear factor-kappa B (NF-κB) and tumor necrosis factor-alpha (TNF-α), which are pivotal in maintaining the prolonged inflammatory phase in diabetic wounds (DWs). Under hyperglycemic conditions, the activation of the Toll-like receptor 4 (TLR4) pathway occurs. Within this pathway, phosphorylated IκB kinase (IKK) activates NF-κB, which then translocates to the nucleus to mediate the expression of various pro-inflammatory cytokine genes. Additionally, the mitogen-activated protein kinase (MAPK) signaling pathways, including extracellular signal-regulated kinase (ERK), c-Jun N-terminal kinase (JNK), and p38 mitogen-activated protein kinase (p38), can activate the transcription factor activator protein 1 (AP-1). The activation of both NF-κB and AP-1 leads to the expression of pro-inflammatory cytokines such as interleukin-6 (IL-6), interleukin-13 (IL-13), and TNF-α, resulting in prolonged inflammation that disrupts the normal wound healing processes.

The inflammatory phase is a vital step leading to hemostasis and employment of the innate immune system, which protects us against the assault of pathogens and helps evacuate dead tissues. However, hyperglycemic conditions stimulate the host tissue to release various pro-inflammatory mediators like cytokines, interleukins, tumor necrosis factor-alpha (TNF-α), interferons, and nuclear factor-kappa B (NF-κB) via Toll-like receptor-4 pathway leading to prolonged inflammation which obstructs the typical phases of wound healing. NF-κB and TNF-α, in turn, provoke the release of various inflammatory mediators via the JNK and ERK pathways as inflammation progresses (Figure 2). NF-κB plays a crucial role in the regulation of genes involved in inflammation, and it is chronically active in many inflammatory diseases like inflammatory bowel disease, arthritis, gastritis, asthma, and atherosclerosis [14,16]. This transcription factor also controls the expression of matrix metalloproteinases [23], which facilitate the secretion of pro-inflammatory mediators such as cytokines, interleukins, and keratinocyte migration [24]. Hence, NF-κB is considered a core transcription regulatory factor for inflammatory and defending processes [25,26,27]. Our investigative drug 5-ASA is proven to inhibit NF-κB activation and TNF-α inhibition. In a study by Bantel H et al. (2000), 5-ASA was shown to inhibit the activation of NF-κB in patients with ulcerative colitis [28]. Furthermore, a study conducted on YAMC cells, a conditionally immortalized murine colon cell line derived from the H-2kb-tsA58 mouse, demonstrated that 5-ASA inhibits TNF-α-mediated effects on the activation of MAP kinase and NF-κB. This suggests that 5-ASA may function as a therapeutic agent by disrupting critical signal transduction events in intestinal cells that are essential for maintaining a chronic inflammatory state [29]. In another study by Raju et al. (2014), male bronchial Balb/C mice were used to investigate the effects of 5-ASA on allergic asthma, which was induced in these mice using ovalbumin (OVA) and alum. After the induction, 5-ASA was administered to the mice, and its impact on inflammatory and oxidative markers was examined. The findings revealed that 5-ASA inhibited IL-6, IL-13, TNF-α, nitrate, nitrite, malonyldialdehyde (MDA), and myeloperoxidase (MPO) levels in a dose-dependent manner [30]. Based on the inhibitory action of 5-ASA on NF-κB and many other inflammatory markers leading to anti-inflammatory activity, we hypothesize that 5-ASA can be used to treat DWs.

### 3.2. 5-ASA and Oxidation

**Figure 3:**
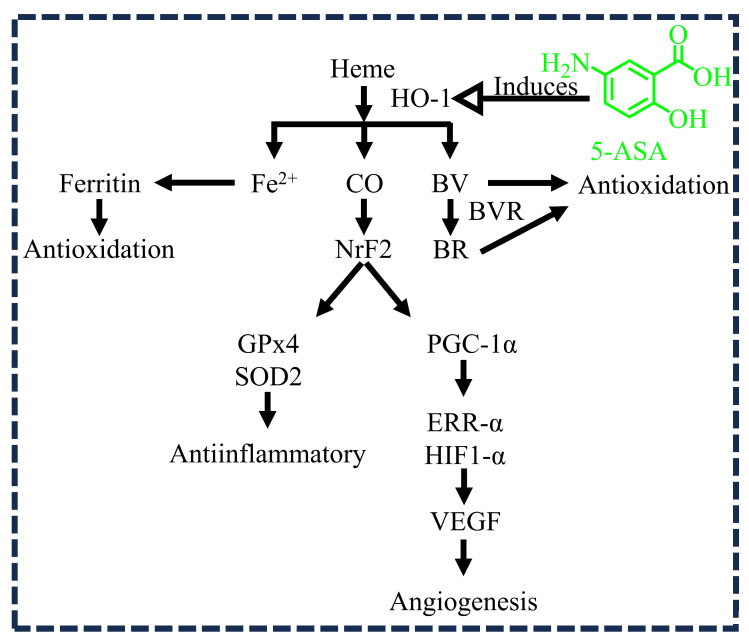
5-aminosalicylic acid (5-ASA) induces the activation of heme oxygenase-1 (HO-1), which leads to various physiological effects, including antioxidation, anti-inflammation, and angiogenesis. The oxidative degradation of heme by HO-1 results in the formation of carbon monoxide (CO), ferrous ion (Fe^2+^), and bilirubin (BR) (converted from biliverdin (BV) by biliverdin reductase (BVR)). The protective effects are observed through the interplay between HO-1 and nuclear factor erythroid 2-related factor 2 (Nrf2), facilitating the anti-inflammatory actions of glutathione peroxidase 4 (GPx4) and superoxide dismutase 2 (SOD2). Furthermore, CO and BR enhance vascular endothelial growth factor (VEGF)-mediated angiogenesis via the peroxisome proliferator-activated receptor-γ coactivator-1α (PGC-1α)-mediated estrogen-related receptor α (ERRα)–hypoxia-inducible factor 1α (HIF-1α) axis. The antioxidant activity is primarily sustained by ferritin, derived from Fe^2+^, as well as by BR and BV.

Heme oxygenase-1 (HO-1) is an antioxidant, anti-inflammatory, and angiogenic enzyme produced by the host epithelium as a defense mechanism. The activated heme oxygenase-1 (HO-1) catalyzes the oxidative degradation of heme into carbon monoxide (CO), ferrous ion, and bilirubin (converted from biliverdin by biliverdin reductase). These products contribute to antioxidation, anti-inflammation, and angiogenesis through different signaling cascade pathways (Figure 3). In general, the impairment of wound healing depends on reactive oxygen species and oxidative stress, which may directly affect the oxidation process. Oxidative stress is a pathogenic mechanism that regulates glycation and hypoxia in the human body. This stress is produced as a result of an imbalance between free radicals and host defensive antioxidants. On the other hand, free radicals are necessary to fight pathogens that cause infections. However, excess free radicals in the body cause damage to tissues, DNA, and proteins [31]. A study by Cavicchi M et al. (2000) investigated the oxidative response in DLD-1 cells, which were cultured in fetal bovine serum. Following the administration of 5-ASA at various concentrations, there was a significant increase in HO-1 induction. The antioxidant properties of the induced HO-1 are likely to contribute to the therapeutic effects of 5-ASA treatments [17]. Therefore, it is reasonable to expect that 5-ASA can produce the enzyme HO-1 (an antioxidant), which regulates oxidative stress in wounds and thereby accelerates the wound healing process.

### 3.3. 5-ASA and TGF-β1 Activation

**Figure 4:**
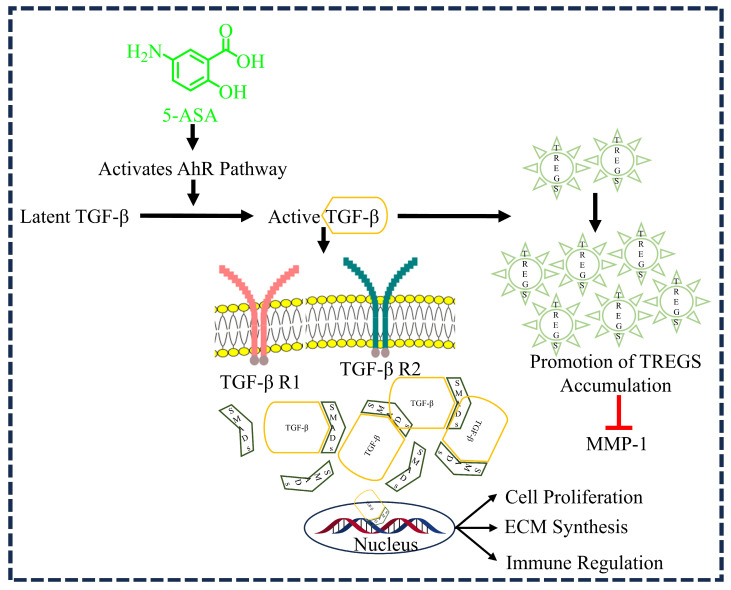
5-aminosalicylic acid (5-ASA) induces cell proliferation, extracellular matrix (ECM) synthesis, and immune regulation via the TGF-β-mediated SMAD-dependent pathway. 5-ASA activates the aryl hydrocarbon receptor (AhR) pathway, which plays a significant role in converting latent transforming growth factor-beta (TGF-β) to its active form. Active TGF-β promotes the accumulation of regulatory T cells (TREGs), contributing to the maintenance of immune homeostasis and suppressing inflammatory conditions. Additionally, TGF-β is crucial for cell proliferation, extracellular matrix (ECM) synthesis, and immune regulation via the SMAD-dependent pathway.

TGF-β is a multifunctional protein that belongs to the family of growth factors and is a critical fibrogenic cytokine. TGF-β, especially β1, is a growth factor involved in several processes of wound healing, such as inflammation, angiogenesis, fibroblast proliferation, collagen synthesis, and extracellular matrix remodeling via the suppressor of mothers against decapentaplegic (SMAD)-dependent pathway (Figure 4) [8,32]. White et al. (1999) conducted an experiment involving rabbit fibroblasts obtained from the knee synovia of young rabbits which demonstrated that TGF-β can antagonize the induction of MMP-1 by phorbol esters in synovial fibroblasts. The results showed that TGF-β inhibited the production of both MMP-1 mRNA and protein. This finding suggests a regulatory role of TGF-β in modulating MMP-1 expression in synovial fibroblasts, thereby preventing the breakdown of collagen fibers [6]. Our investigated drug, 5-ASA, has been proven to bind to TGF-β, specifically β1, which in turn activates regulatory T cells (TREGs). These cells have shown potential in exerting anti-inflammatory effects, suppressing inflammatory diseases, preventing autoimmune conditions, and promoting antibody production (Figure 4) [22]. TREGs perform suppressive functions in disparate tissue environments and against inflammatory conditions. Kyoto et al. (2017) investigated the impact of 5-ASA on TREG frequencies in the colons of different mouse models, including wild-type mice, AhR-deficient mice (AhR−/−), and bone marrow–chimeric mice lacking AhR in hematopoietic cells (BM-AhR−/−). They also explored the effects of 5-ASA on TGF-β expression in the colon. The study found that 5-ASA treatment in wild-type mice increased the accumulation of TREGs in the colon and upregulated the aryl hydrocarbon receptor (AhR) target gene Cyp1A1. This effect was not seen in AhR−/− or BM-AhR−/− mice. Furthermore, 5-ASA facilitated the in vitro differentiation of naive T cells into TREGs alongside AhR activation. In wild-type mice treated with 5-ASA, there was an increase in the active form of TGF-β in the colon, dependent on AhR. Blocking TGF-β signaling suppressed mesalamine-induced TREG formation in the colon. These findings propose a novel anti-inflammatory mechanism for 5-ASA in colitis treatment: the induction of TREGs in the colon via the AhR pathway, followed by TGF-β activation [22]. Considering the importance of TGF-β1 in chronic wounds [33,34], targeting TGF-β1 might be a logical rationale encouraging the use of 5-ASA in the treatment of DWs.

### 3.4. 5-ASA and PPAR-γ Activation

PPAR-γ is a critical regulator of glucose and lipid metabolism, as it can act as a transcription factor that is responsible for stimulating protein synthesis in many processes (energetic metabolism, proliferation, and cellular differentiation). PPAR-γ is known as the glitazone receptor, is activated by many transcription factors, and plays a significant role in the regulation of cell inflammation and cell proliferation. Ligand activation of PPAR-γ can downregulate genes encoding inflammatory molecules, inflammatory cytokines, growth factors, proteolytic enzymes, adhesion molecules, chemotactic factors, and atherogenic factors [35]. From these studies, it was clear that PPAR-γ agonists may play a significant role in the inhibition and infiltration of macrophages through MMP inhibition, chemokine modulation, cell signal transduction, and neovascularization, and possess antifibrotic properties [36]. Thiazolidinediones are classical PPAR-γ agonists currently used as insulin-sensitizing agents in the treatment of type 2 diabetes [20].

**Figure 5:**
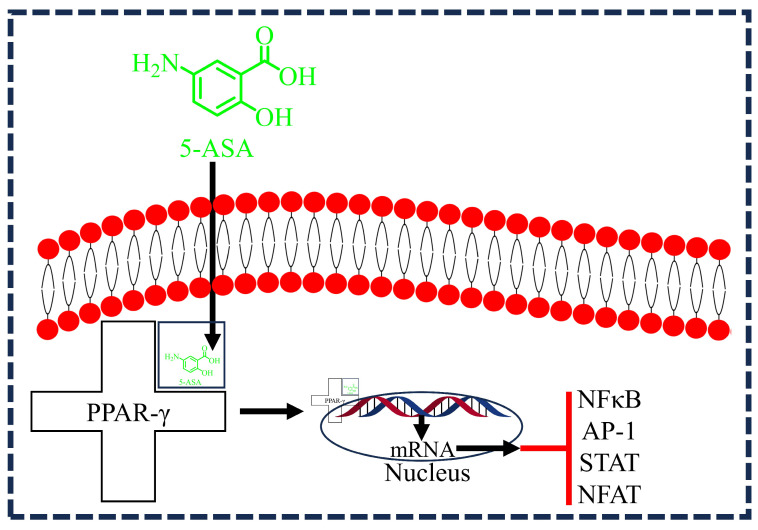
Illustration detailing the mechanism of action of 5-aminosalicylic acid (5-ASA) in diabetic wound (DW) therapy. After entering the cell, 5-ASA binds to PPAR-γ, forming a complex that translocates to the nucleus. This complex enhances mRNA transcription, leading to the inhibition of nuclear factor-kappa B (NFκB), activator protein 1 (AP-1), signal transducer and activator of transcription (STAT), and nuclear factor of activated T cells (NFAT). These actions collectively contribute to the anti-inflammatory effects of 5-ASA.

5-ASA also acts as a PPAR-γ agonist and induces its activation and expression in epithelial cells. After entering the cell, 5-ASA binds to PPAR-γ, forming a complex that translocate to the nucleus. This complex enhances mRNA transcription, leading to the inhibition of NFκB, AP-1, signal transducer and activator of transcription (STAT), and nuclear factor of activated T cells (NFAT). These actions collectively contribute to the anti-inflammatory effects of 5-ASA (Figure 5) [37]. A study by Rousseaux et al. (2015) explored the effects of 5-ASA on PPAR-γ activation in the context of colitis. The researchers used male 129/Sv mice, inducing severe colitis through intrarectal administration of 2,4,6-trinitrobenzene sulfonic acid (TNBS), a model that closely mimics human inflammatory bowel diseases (IBDs). To investigate the role of 5-ASA as a PPAR-γ activator, they analyzed its impact on confluent 3T3-L1 preadipocytes and HT-29 cells. The results showed a three-fold induction of PPAR-γ mRNA in HT-29 cells treated with 5-ASA. The activation process of PPAR-γ involves its translocation to the nucleus, conformational changes, recruitment of coactivators, and binding to peroxisome-proliferator response elements (PPRE), all of which were examined in the study to understand the comprehensive effects of 5-ASA. Further validation was conducted using organ cultures of right colonic biopsies from 12 patients, including non-IBD patients and those with Crohn’s disease or ulcerative colitis. The findings confirmed that 5-ASA interacts with PPAR-γ as its key receptor, mediating significant therapeutic effects in the colon [21]. Considering the functional importance of PPAR-γ in wound healing progression, 5-ASA may be beneficial for the treatment of DWs.

### 3.5. 5-ASA and Re-Epithelialization

Epithelial cell migration and cell proliferation are the fundamental processes that occur in phase 3 (the proliferation phase) of wound healing. During the proliferation phase, the cells proliferate; eventually, granulation tissue replaces the wound matrix formed during hemostasis. The granulation tissue formed consists of macrophages, blood vessels, and fibroblasts in complex with collagen bundles, which partially recovers the functional structure of the wounded skin [38]. Epithelial cell migration favors wound debridement and the removal of nonviable tissue material (necrotic tissue), which acts as fuel for infection and impedes wound healing. Baumgart et al. (2013) investigated the effects of 5-ASA on mucosal healing using the non-transformed small intestinal epithelial cell line IEC-6 in vitro. Their study employed an in vitro wounding model and MTT assays to assess cell migration and proliferation, respectively. Additionally, Trypan blue exclusion and flow-cytometry-based cell cycle analysis were utilized to evaluate epithelial cell viability following treatment with 5-ASA. Clinically relevant concentrations of 5-ASA demonstrated a significant, dose-dependent enhancement of epithelial cell migration and proliferation. Specifically, pharmacological doses of 100 µg/mL 5-ASA led to approximately two-fold increases in these processes. Importantly, the stimulatory effects of 5-ASA on IEC-6 cell proliferation and migration were not affected by neutralizing antibodies against TGF-β, indicating an independent mechanism from TGF-β. Furthermore, viability tests and cell cycle analysis showed no evidence of toxicity or apoptosis at pharmacological concentrations of 5-ASA in IEC-6 cells. These findings suggest that 5-ASA promotes rapid mucosal integrity restoration by directly stimulating epithelial restitution and proliferation, complementing its established role in anti-inflammatory activity within the intestinal cascade [19]; therefore, it could be used for the treatment of DWs.

To further support our hypothesis, we have summarized (Table 1) the existing literature on how 5-ASA exerts its pharmacological effects relevant to the pathophysiology of DWs.

## 4. Implications

Innovative Wound Treatment: Researchers are investigating the potential of using 5-aminosalicylic acid (5-ASA) as a treatment for diabetic wounds, which could represent a groundbreaking approach in wound care for individuals with diabetes.

Comprehensive Healing Potential: The multifactorial nature of the effects of 5-ASA suggests that it may offer a range of therapeutic benefits, including anti-inflammatory, antimicrobial, and tissue regeneration properties. This comprehensive approach could significantly enhance the healing process for diabetic wounds.

Promising Therapeutic Avenue: Initial findings indicating the efficacy of 5-ASA in improving wound healing outcomes hold promise for advancing diabetic wound therapy. If validated through further research, this could offer a much-needed solution for addressing the challenges associated with diabetic wound management.

Exploring Untapped Opportunities: The characterization of 5-ASA’s potential as “unexplored” underscores the novelty of this research endeavor. By delving into previously uncharted territory, this study aimed to uncover new insights and therapeutic strategies for diabetic wound care.

Translation into Clinical Practice: Successful validation of the efficacy of 5-ASA in preclinical studies could pave the way for its translation into clinical practice. If proven effective and safe in human trials, 5-ASA could emerge as a valuable therapeutic option for improving wound healing outcomes in diabetic patients, thereby addressing a critical unmet medical need.

## 5. Conclusions

In conclusion, 5-ASA exhibits promising anti-inflammatory, antioxidant, and re-epithelialization properties relevant to DWs. Due to its chemical structure, efficient and stabilized formulations of 5-ASA are essential for effective topical delivery. Our hypothesis suggests that 5-ASA, via activation of PPAR-γ and TGF-β and inhibition of the NFκ-B pathway, could benefit DW patients by addressing inflammation, circulatory issues, impaired tissue regeneration, and neuropathy. However, further research is necessary to confirm its effects on insulin sensitivity and resistance, which are crucial for managing diabetes-related complications. Preclinical studies with optimized formulations are required to validate 5-ASA’s potential in treating DWs.

## 6. Future Directions and Technical Roadmap

Our hypothesis that 5-ASA has multifactorial potential in DW therapy necessitates a systematic and comprehensive approach for validation.

In vitro studies: To begin, in vitro studies will identify the molecular pathways through which 5-ASA promotes wound healing in diabetic patients. Experiments will evaluate its anti-inflammatory effects on markers such as TNF-α, IL-6, and IL-1β. Additionally, studies on cell proliferation and migration in keratinocytes and fibroblasts will provide insights into how 5-ASA facilitates wound repair.

In vivo studies: Next, in vivo research using diabetic animal models will assess the therapeutic efficacy of 5-ASA in wound healing. Key outcomes will include wound closure rates, histological analysis, and evaluation of collagen deposition and angiogenesis. Employing advanced imaging techniques like multiphoton microscopy will allow for real-time observation of the wound healing process.

Omics approaches: Utilizing omics technologies such as transcriptomics and proteomics will provide a detailed understanding of the molecular alterations induced by 5-ASA treatment. These high-throughput methods will help identify new pathways and biomarkers associated with the therapeutic effects of 5-ASA.

Clinical trials: Finally, conducting clinical trials involving diabetic patients with chronic wounds will validate the preclinical findings. These trials should be designed as randomized controlled trials to rigorously evaluate the safety and efficacy of 5-ASA in improving wound healing outcomes in this patient group.

## Figures and Tables

**Table 1:** Overview of studies exploring the role of 5-ASA in different phases of wound healing.

Drug/ Formulation	Pharmacological Effect	Biomarkers	Type of Study Conducted	References
5-ASA-bound nanoparticles	Inflammation	Clinical activity score (CAS), colon weight/length index, myeloperoxidase (MPO)	Preexistent colitis model in mice	[39]
5-ASA and chitosan combination	Inflammation	Clinical activity score (CAS), colon weight/length index, myeloperoxidase (MPO), alkaline phosphatase (ALP), TNF-α, IL-6, and NF-κB, p65	Colitis model in male Swiss/CD-1 mice	[40]
5-ASA in combination with acacia and guar gum	Inflammation	Disease activity index, colon weight/length ratio, IL-1β, NF-κB, p65, TNF-α, and IL-6	Colitis model in male Swiss/CD-1 mice	[41]
5-ASA and hyaluronic acid combination	Inflammation	Clinical activity index, MPO, TNF-α, IL-6, and IL-1β	Colitis model in male Swiss/CD-1 mice	[42]
5-ASA and chitosan microspheres	Inflammation	Cell viability and expression of mRNA levels	Caco-2 cell lines	[43]
5-ASA	Inflammation	IL-6, IL-8, COX-2, nitric oxide, glycosaminoglycan, and anabolic genes (aggrecan (ACAN), alpha-1 chain of type II collagen (COL2A1), proteoglycan 4 (PRG4), cartilage oligomeric matrix protein (COMP))	In vitro (chondrocyte pellets) and ex vivo (osteochondral explants) human inflammatory osteoarthritis models	[44]
5-ASA	Inflammation	NO, IL-6, induced nitric oxide synthase (iNOS), c-Jun N-terminal kinases (JNKs), p38, and NF-κB	LPS-induced murine macrophages	[45]
5-ASA–silicon oxide nanoparticles	Inflammation	MPO, IL-6, and TNF-α	BALB/c colitis model	[46]
5-ASA in combination with hyaluronic acid	Inflammation	MPO, COX-2, TNF-α, IL-1β, and IL-6	TNBS-induced colitis rat model	[47]
5-ASA gelatin-coated nanoparticles	Inflammation	TNF-α, IL-1β, COX-2, and iNOS	Dextran sodium sulfate-induced colitis murine model	[48]
5-ASA	Oxidation	Free radicals such as hydroxyl, haloperoxyl, one-electron oxidizing, lipid peroxyl, glutathiyl, superoxide, tryptophany	Nanosecond pulse radiolysis technique coupled with transient spectrophotometry has been used for in situ generation of free radicals and to follow their reaction pathways	[49]
5-ASA	Oxidation	ROS (O_2_^•−^, H_2_O_2_, ^1^O^2^, ROO•, and HOCl) and reactive nitrogen species (^•^NO and ONOO^−^)	Chemical scavenging validated test	[50]
5-ASA in combination with cyanidin-3-glucoside (Cy3glc)	Oxidation	ROS and NO	LPS-activated macrophage line	[51]
5-ASA	Oxidation	ROS species for oxidative DNA damage	Diversion colitis model in experimental Wistar rats	[52]
5-ASA and ascorbic acid	Oxidation	Vitamin E consumption, oxygen consumption and formation of conjugated dienes	Lipid peroxidation (thermal decomposition of Azo compounds) in phosphatidylcholine (PC) liposomes as a model	[53]
5-ASA	Oxidation	ROS and catalase	Oxidant-induced cell signaling pathways in HT-29 cells and IECs from mice	[54]
5-ASA and vitamin E	Oxidation	Change in body weight and lactate dehydrogenase activity	Acrylamide-induced kidney injury in Wistar rat model	[55]
5-ASA and lycopene	Oxidation and inflammation	Myeloperoxidase (MPO), malondialdehyde (MDA), and superoxide dismutase (SOD)	Colitis model in iodoacetamide rat	[56]
5-ASA	Inflammation and PPAR-γ activation	Oxygen consumption, *E. coli* growth	Dextran sulfate sodium (DSS)-induced colitis murine model	[57]
5-ASA	Inflammation and PPAR-γ activation	PPAR-γ expression, β-actin, and MPO	2,4,6-trinitrobenzenesulphonic acid (TNBS)-induced colitis murine model	[21]
5-ASA	Inflammation and PPAR-γ activation	IFN-γ, NF-κB, STAT-1 and -3, SOCS-1 and -3	10 Gy γ-irradiation (Co source)-induced colitis rat model	[58]
5-ASA and n-3 ploy unsaturated fatty acids	Inflammation and PPAR-γ activation	NF-κB, COX-2, and leukotriene-B_4_	TNBS-induced colitis rat model	[59]
5-ASA	Inflammation, oxidation, and proliferation	ROS, MTT assay, cell apoptosis assay, caspase-3 activity, SOD2 and wound healing assay	Indomethacin-induced injury in IEC-6 cell line of rats	[60]
5-ASA in combination with azathioprine	Inflammation, oxidation, and proliferation	ROS and senescence-associated β-galactosidase activity, cell cycle analysis, BrdU incorporation assay, TNF-α	T-84 cell lines and small intestinal large bowel organoids from C57BL/6J wild-type and IL-10−/− (IL-10 KO) mice	[61]
5-ASA pluronic lecitin organogel	Cell proliferation and migration	MTT assay, cell migration	Full thickness excision wound rat model	[6]
5-ASA	Cell proliferation and migration	MTT assay, migration assay	IEC-6 in vitro wounding model	[19]
5-ASA	Angiogenesis	Expression of VEGF, endostatin, angiostatin, TNF-α, and MMP-2 and -9	Iodoacetamide-induced ulcerative colitis rat model	[62]

## Data Availability

Not applicable.

## List of Abbreviations

**Abbreviation**	**Full Form**
DM	Diabetes mellitus
DW	Diabetic wound
DFU	Diabetic foot ulcers
5-ASA	5-aminosalicylic acid
PPAR-γ	Peroxisome proliferator-activated receptor gamma
TGF-β	Transforming growth factor beta
ECM	Extracellular matrix
MMP	Matrix metalloproteinase
NF-κB	Nuclear factor-kappa B
TNF-α	Tumor necrosis factor-alpha
HO-1	Heme oxygenase-1
TREG	Regulatory T cell
MPO	Myeloperoxidase
CAS	Clinical activity score
PC	Phosphatidylcholine
ALP	Alkaline phosphatase
COMP	Cartilage oligomeric matrix protein
MDA	Malondialdehyde
SOD	Superoxide dismutase
